# Transcriptomic profiling of ceftriaxone-tolerant phenotypes of *Neisseria gonorrhoeae* reveals downregulation of ribosomal genes — a pilot study

**DOI:** 10.1128/spectrum.01207-25

**Published:** 2025-07-07

**Authors:** Sheeba Santhini Manoharan-Basil, Margaux Balduck, Zina Gestels, Said Abdellati, Tessa De Block, Chris Kenyon

**Affiliations:** 1 Department of Clinical Sciences, Institute of Tropical Medicine Antwerp, Antwerp, Belgium; 2 Clinical Reference Laboratory, Institute of Tropical Medicine Antwerp, Antwerp, Belgium; 3 Department of Medicine, University of Cape Town, Cape Town, South Africa; Central Texas Veterans Health Care System, Temple, Texas, USA

**Keywords:** tolerant, RNA-seq, ceftriaxone, WHO P, *Neisseria gonorrhoeae*

## Abstract

Antibiotic tolerance is associated with failure of antibiotic treatment and accelerates the development of antimicrobial resistance. The molecular mechanisms underlying antimicrobial tolerance remain poorly understood. Tolerant bacteria can slow metabolism by extending the lag phase without altering antimicrobial susceptibility. We recently induced ceftriaxone (CRO) tolerance in the *Neisseria gonorrhoeae* reference strain WHO P. In the current study, we characterized the transcriptomic profiles of these CRO-tolerant phenotypes. To induce tolerance, WHO P strains were grown under 3-h intermittent CRO exposure (10× the MIC), followed by overnight growth in gonococcal (GC) broth for seven consecutive days, with cultures maintained in sextuplicate. Two control cultures were maintained without CRO exposure. The tolerance and CRO susceptibility of the isolates were assessed using a modified tolerance disc (TD) test. Total RNA was isolated from tolerant isolates (n = 12) and control (n = 3) strains, followed by Ribo depletion, Illumina Library preparation, and sequencing. Transcriptomic analysis revealed no differentially expressed genes after 1 day of CRO exposure. However, after 3 days of CRO exposure, 13 genes were found to be significantly downregulated, including tRNA-Ser (C7S06_RS03100) and tRNA-Leu (C7S06_RS04945) and ribosomal RNA genes (16S and 23S rRNA). Following 7 days of exposure, 51 genes were differentially expressed, with most downregulated, such as SecB (Protein-export chaperone SecB) and tRNA-Ser (C7S06_RS01850) and the 16S and 23S ribosomal RNA genes. The development of CRO-tolerance in *N. gonorrhoeae* was associated with the downregulation of various ribosomal genes and associated genes, reflecting a potential mechanism for bacterial survival under antibiotic stress.

**IMPORTANCE:**

Antibiotic tolerance allows some bacteria to survive antibiotic treatment, contributing to treatment failure and creating conditions that promote resistance. In this study, we showed that *Neisseria gonorrhoeae*, the bacteria that causes gonorrhea, can become tolerant to ceftriaxone—the last-line treatment used. By repeatedly exposing the bacteria to high doses of ceftriaxone, we observed the development of tolerance over several days. Using transcriptomic analysis, we found that tolerant bacteria consistently reduced the activity of genes involved in protein synthesis, including ribosomal RNAs and transfer RNAs. This suggests that *N. gonorrhoeae* may survive antibiotic stress by entering a low-metabolic state that makes the antibiotic less effective. These findings highlight a survival mechanism that does not rely on genetic resistance. Understanding this tolerance response is vital for improving current treatment approaches and could inform the development of new strategies to prevent antibiotic failure in gonorrhea and other infections.

## INTRODUCTION

Antibiotic tolerance poses a significant challenge in clinical settings and is increasingly recognized as a significant factor associated with treatment failure and recurrent infections (1). Unlike resistance, where there is an increase in the minimum inhibitory concentration (MIC) of antibiotics (2–4), tolerance enables bacteria to acquire the ability to survive transient exposure to high antibiotic concentrations through adaptation without an increase in the MIC. One way they do this is by slowing down their metabolism by extending the lag phase (5–11). The emergence of tolerance has been shown to facilitate the emergence of antimicrobial resistance (AMR) (11). Understanding the mechanisms responsible for tolerance could thus assist with the development of not only novel treatment approaches to prevent treatment failures but also the emergence of AMR (11, 12).

Antibiotic tolerance has been observed in numerous bacterial species. Although the mechanisms underpinning tolerance vary between different species, they have frequently been found to include alterations in gene expression, changes in cell envelope properties, metabolic slowdown, activation of stress response pathways, and the formation of persister cells (13, 14). Antibiotic tolerance has been reported in clinically relevant pathogens, such as *Staphylococcus aureus*, *Escherichia coli*, *Pseudomonas aeruginosa*, *Streptococcus pneumoniae*, and *Mycobacterium* spp. (13, 15–17). For instance, *S. aureus* can survive antibiotic exposure by forming persister cells that regrow, leading to recurrent infections (18), while *M. tuberculosis* enters a dormant state characterized by changes, such as a dramatic downregulation of rRNA and ribosomal protein synthesis (19, 20). Upregulation of the *relA* gene was seen in ciprofloxacin-tolerant *S. aureus* (21), and deletion of the sodium-proton antiporter gene *nhaA* resulted in downregulated metabolism and upregulated stress responses in *E. coli* (22). In *Mycobacterium abscessus*, the cytochrome bd-type quinol oxidase contributes to clofazimine tolerance (23). These diverse mechanisms highlight the complexity of antibiotic tolerance across bacterial species and underscore the need for species-specific approaches in addressing this clinical challenge.

Resistance to ceftriaxone, the first-line treatment for gonorrhoea—a sexually transmitted infection (STIs) caused by the Gram-negative bacterium *Neisseria gonorrhoeae*—has become a significant concern. We have shown that tolerance to ceftriaxone can be induced in *N. gonorrhoeae* through cyclic antibiotic exposure (24). Mutations in four genes that may contribute to ceftriaxone tolerance in *N. gonorrhoeae*, including mutation in the enolase (*eno*) gene, have been identified (25). Of interest, the prevalence of antibiotic tolerance in *N. gonorrhoeae* varies by anatomical site, with tolerance to azithromycin being more common in anorectal than urogenital infections (26). The intermittent ceftriaxone exposure model used in this study was designed to mimic clinical scenarios where gonococci are exposed to fluctuating concentrations of ceftriaxone. Such conditions may arise due to incomplete treatment adherence or reduced antimicrobial penetration in anatomical niches, such as the rectum, cervix, or pharynx. This is supported by clinical reports of recurrent gonococcal infections despite treatment with ceftriaxone-susceptible strains, suggesting a role for phenotypic tolerance rather than resistance. In one such case, repeated treatment failures were hypothesized to involve ceftriaxone-tolerant subpopulations potentially due to biofilm-like environments (27).

Gene expression studies have revealed several transcriptional regulators involved in bacterial antibiotic tolerance. For instance, *marA*, *soxS,* or *robA* systems in *Escherichia coli* have been implicated in mediating the induction of multi-drug tolerance (28, 29). No transcriptomic studies are available that provide insights into the mechanisms underlying ceftriaxone tolerance in *N. gonorrhoeae*.

This study aimed to provide the transcriptomic profile of *in vitro*-generated ceftriaxone tolerance strains of *N. gonorrhoeae* using RNA-Seq. We aimed to identify gene expression associated with the ceftriaxone-tolerant phenotype.

## MATERIALS AND METHODS

### Induction of CRO tolerance, assessment of tolerance, and CRO susceptibility

Previously, we induced ceftriaxone (CRO) tolerance in a highly CRO susceptible *N. gonorrhoeae* reference strain, WHO P, with a CRO MIC of 0.004 µg/mL (24). Briefly, the overnight culture of WHO *P* reference colonies was suspended in gonococcal (GC) broth, and the turbidity was adjusted to 0.5–1.0 McFarland. These suspensions were then exposed to a high concentration of CRO at 10 times the MIC (0.04 µg/mL) for 3 h in a cyclic manner. After each cycle, the cultures were resuspended in GC broth containing 0.04 µg/mL CRO, followed by washing the bacterial cells to remove antibiotics, and were incubated overnight at 36°C in a 6% CO_2_ incubator. The experiment was conducted over seven consecutive days with six biological replicates and two control replicates that did not receive antibiotic exposure. The CRO MIC was determined after each exposure cycle using a gradient E-test, and samples were stored at −80°C in skim milk with 30% glycerol.

To detect tolerant phenotypes, a modified tolerance disc (TD) test was used, wherein the colonies were inoculated on BD^TM^ Columbia Agar with 5% Sheep Blood, followed by incubation and sequential exposure to discs with CRO (antibiotic discs with CRO concentration of 0.04 µg/mL) and discs with GC broth (nutrient discs) (24). Tolerant colonies that emerged after 48 h were further inoculated on fresh blood agar plates, harvested in RNA Shield (Zymo Research, Netherlands), snap-freezed, and outsourced to Eurofins Genomics (Germany) for RNA isolation and sequencing.

### RNA sequencing and bioinformatic analyses

A total of 15 isolates were subjected to RNA-Seq, comprising 12 ceftriaxone-tolerant isolates and three control isolates that were passaged in parallel without CRO exposure (Table 1). The tolerant isolates included three isolates each from three time points (after days 1, 3, and 7 of CRO exposure) and four independent lineages (Table 1). Total RNA was isolated from a total of 15 isolates, followed by ribodepletion to remove ribosomal RNA. The Stranded TruSeq RNA Library Preparation Kit was used for cDNA library preparation. The library was then sequenced on a NextSeq6000, v2, 1 × 150 bp platform (Illumina Inc). Data analysis was conducted using CLC Genomics Workbench v22 (Clcbio, Denmark), according to reference 30. Differential gene expression (DGE) analysis was performed to identify genes that showed significant differences in expression using Benjamini-Hochberg multiple testing correction using a false discovery rate of 0.05. The analysis also included controlling for time points across all groups to assess the differential expression due to CRO exposure.

**TABLE 1:** Evolution of ceftriaxone tolerance in the samples assessed^*c*^

CRO exposure [in days]^*b*^								
Days (d)	d0	d1	d2	d3	d4	d5	d6	d7
Control 1	–^*a*^	10.2	–	12.1	–	–	–	16.1
Control 2	–	–	–	–	–	–	–	–
Lineage 1	–	10.3^T^	11.3-2.3 ^T^	12.3.3^T^	13.3.3 ^T^	14.3.3 ^T^	15.3-2.3 ^T^	16.3–2.3 ^T^
Lineage 2	–	10.4^T^	11.4-2.3 ^T^	12.4.3^T^	13.4.3 ^T^	14.4.3 ^T^	15.4-2.3 ^T^	16.4–2.3 ^T^
Lineage 3	–	–	11.5-2.3 ^T^	–	–	–	–	16.5–2.3 ^T^
Lineage 4	–	–	–	12.6.3 ^T^	–	–	–	16.6–2.3 ^T^
Lineage 5	–	10.7^T^	11.7-2.3 ^T^	12.7.3 ^T^	13.7.3 ^T^	14.7.3 ^T^	15.7.3 ^T^	16.7–2.3 ^T^
Lineage 6	–	10.8 ^T^	11.8-2.3 ^T^	12.8.3^T^	–	–	–	16.8–2.3 ^T^

^*a*^ - Samples stored/collected.

^*b*^ T- Tolerant isolates.

^*c*^ The experiment was conducted over seven consecutive days with six biological replicates and two control replicates of *N. gonorrhoeae* WHO P. While lineages 1–6 were exposed to daily ceftriaxone, the controls were exposed to identical conditions with the exception of not being exposed to ceftriaxone daily. The isolates that were subjected to RNA-Seq are colored in gray.

## RESULTS

**Fig 1:**
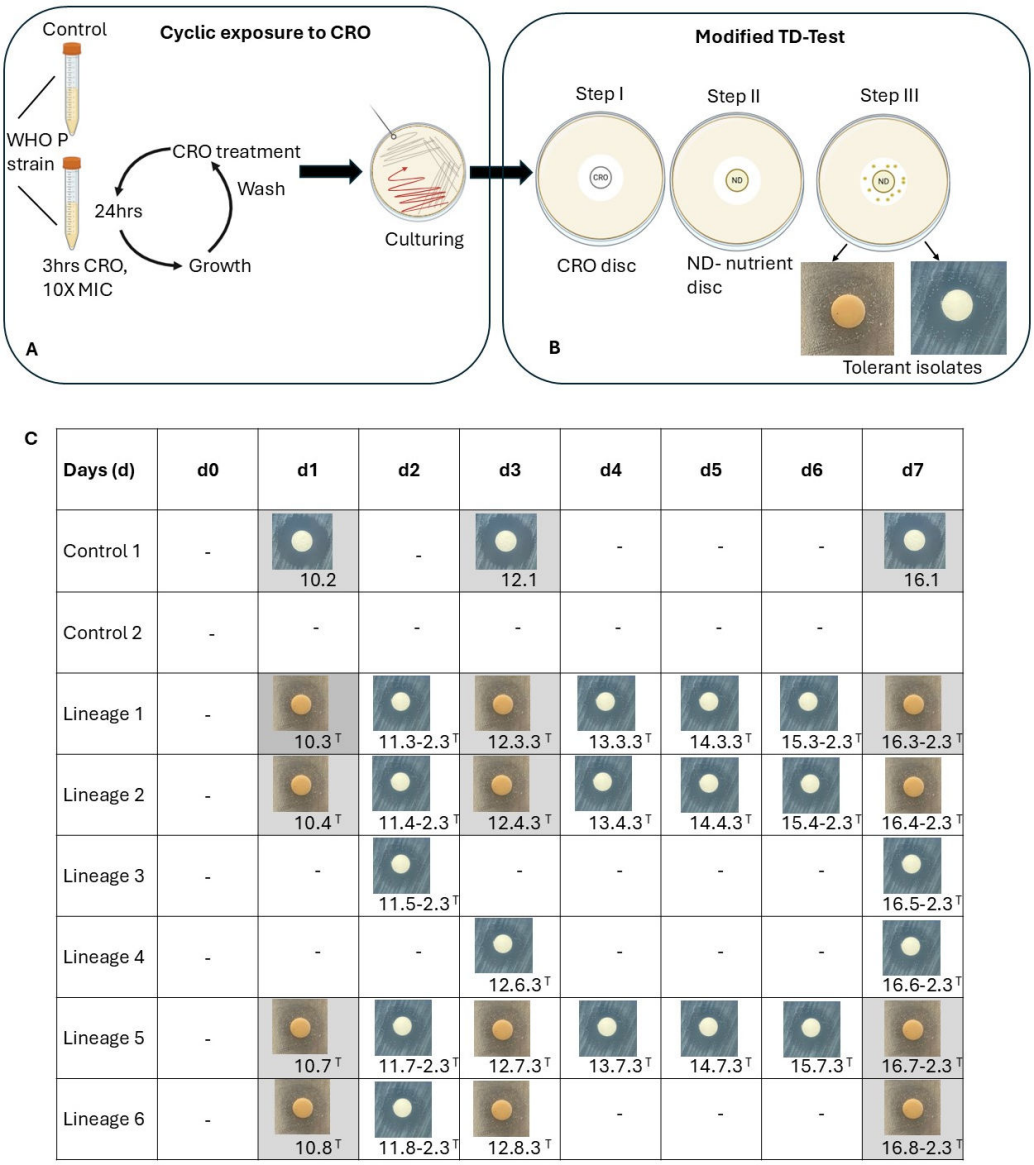
Induction of ceftriaxone (CRO) tolerance in *Neisseria gonorrhoeae* WHO P reference strain using a cyclic exposure model and detection of CRO-tolerant isolates using modified tolerance disc (TD) test. (**A**) Schematic representation of the experimental design. CRO tolerance was induced by exposing the WHO P reference strain to cyclic treatment with 10 × MIC ceftriaxone for 3 h daily, followed by growth in fresh GC broth for 24 h. This cycle was repeated over seven consecutive days. Cultures were maintained in sextuplicate, with two control cultures passaged in parallel without antibiotic exposure. (**B**) Modified TD test protocol. Step I: Colonies were exposed to a CRO disc (0.008 µg) for 18 h; Step II: After initial observation of the zone of inhibition, the CRO disc was replaced with a nutrient disc (ND) containing GC broth and incubated overnight; Step III: Addition of GC broth to the ND disc and emergence of tolerant isolates around the ND disc was assessed after 48 h. Representative images of tolerant colonies are shown. (**C**) Timeline and photographic documentation of isolate recovery over 7 days. Tolerant isolates are denoted with a superscript “T.” The samples that were subjected to RNA sequencing are shown in gray.

In this study, a comparative transcriptomic analysis was carried out to identify differentially expressed genes (DEGs) between control (*n* = 3) and tolerant isolates (*n* = 12) across four lineages and three time points (Fig. 1). The experimental conditions were as follows (Table 1, Fig. 1):

1. Condition 1: 10.2 (control sample) vs 10.3, 10.4, 10.7, and 10.8 (tolerant samples) isolates from day 1 CRO-exposure
2. Condition 2: 12.1 (control sample) vs 12.3.3, 12.4.3, 12.7.3, and 12.8.3 (tolerant samples) isolates from day 3 CRO-exposure
3. Condition 3: 16.1 (control sample) vs 16.3–2.3, 16.4–2.3, 16.7–2.3, and 16.8–2.3 (tolerant samples) isolates from day 7 CRO-exposure
4. Condition 4: all the control samples (*n* = 3) vs all the tolerant samples (*n* = 12).

In condition 1, no DEGs were identified.

**Fig 2:**
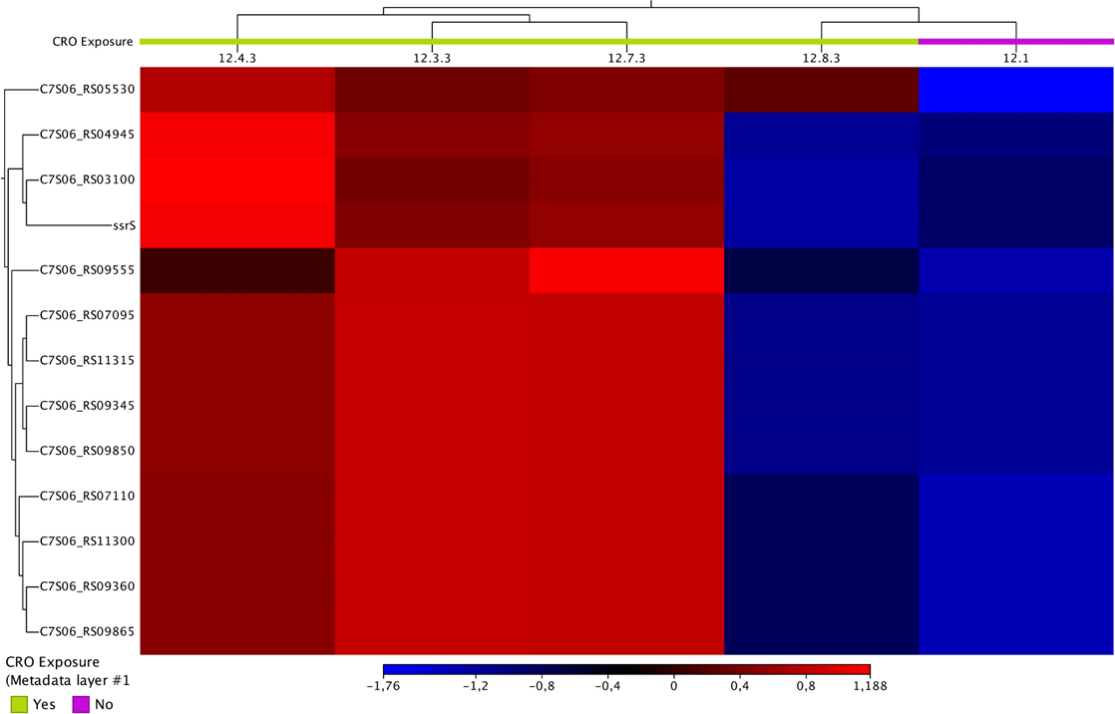
Heatmap of differentially expressed genes in condition 2 showing the expression levels of all 13 DEGs across the CRO-tolerant and control isolates

In condition 2, we identified 13 DEGs, which were significantly downregulated in the tolerant isolates (Fig. 2 and Table 2). Notable DEGs included C7S06_RS03100 (tRNA-Ser) and C7S06_RS04945 (tRNA-Leu), with log_2_ fold changes of −7.40 and −8.83, respectively. Several ribosomal RNA genes, including four copies each of 23S ribosomal RNA (C7S06_RS07095, C7S06_RS09345, C7S06_RS09850, C7S06_RS11315) and 16S ribosomal RNA (C7S06_RS07110, C7S06_RS09360, C7S06_RS09865, C7S06_RS11300) and *ssrS* (6S RNA), showed significant downregulation (Fig. 2 and Table 2).

**TABLE 2:** List of differentially expressed genes for conditions 1 to 4^*a*^

Conditions	Samples	Name	Product	Log2 fold change	*P*-value	FDR *P*-value
Condition 1	10.2 vs 10.3, 10.4, 1.0.7, 10.8	NA	NA	NA	NA	NA
Condition 2	12.1 vs 12.3.3, 12.4.3, 12.7.3, 12.8.3	C7S06_RS03100	tRNA-Ser	−7.40	0.00	0.00
		C7S06_RS04945	tRNA-Leu	−8.83	0.00	0.00
		C7S06_RS05530	Helix-turn-helix domain-containing protein	−6.99	0.00	0.00
		C7S06_RS07095	23S ribosomal RNA	−7.36	0.00	0.00
		C7S06_RS07110	16S ribosomal RNA	−8.49	0.00	0.00
		C7S06_RS09345	23S ribosomal RNA	−7.37	0.00	0.00
		C7S06_RS09360	16S ribosomal RNA	−8.51	0.00	0.00
		C7S06_RS09555	Hypothetical protein	−3.17	0.00	0.00
		C7S06_RS09850	23S ribosomal RNA	−7.38	0.00	0.00
		C7S06_RS09865	16S ribosomal RNA	−8.56	0.00	0.00
		ssrS	6S RNA	−4.01	0.00	0.02
		C7S06_RS11300	16S ribosomal RNA	−8.57	0.00	0.00
		C7S06_RS11315	23S ribosomal RNA	−7.34	0.00	0.00
Condition 3	16.1 vs 16.3.3, 16.4.3, 16.7-2.3, 16.8-2.3	secB	Protein-export chaperone SecB	−1.85	0.00	0.01
		C7S06_RS01160	2,3-Diphosphoglycerate-dependent phosphoglycerate mutase	−1.29	0.00	0.04
		C7S06_RS01850	tRNA-Ser	−8.00	0.00	0.00
		C7S06_RS02470	IS1595 family transposase	−3.03	0.00	0.00
		htpX	Protease htpX	−1.92	0.00	0.02
		C7S06_RS02975	tRNA-Gln	−5.72	0.00	0.00
		cysT	sulfate ABC transporter permease subunit CysT	1.46	0.00	0.01
		C7S06_RS03025	Isoprenylcysteine carboxyl methyltransferase family protein	−1.31	0.00	0.01
		C7S06_RS03100	tRNA-Ser	−15.02	0.00	0.03
		C7S06_RS03105	tRNA-Ser	−7.49	0.00	0.02
		C7S06_RS03165	Hypothetical protein	−6.49	0.00	0.00
		C7S06_RS03180	Hypothetical protein	−3.59	0.00	0.00
		C7S06_RS03675	carbonic anhydrase family	−1.83	0.00	0.01
		C7S06_RS04010	Alpha-hydroxy-acid oxidizing protein	−2.16	0.00	0.00
		glnD	[protein-PII] uridylyltransferase	1.14	0.00	0.02
		C7S06_RS04945	tRNA-Leu	−16.20	0.00	0.01
		C7S06_RS04950	tRNA-Ser	−8.19	0.00	0.00
		C7S06_RS05295	Serine hydroxymethyltransferase	−1.45	0.00	0.01
		C7S06_RS05355	Sulfate ABC transporter substrate-binding protein	1.21	0.00	0.04
		C7S06_RS05905	protein-disulfide reductase DsbD	−1.94	0.00	0.04
		C7S06_RS06055	Hypothetical protein	−2.92	0.00	0.00
		C7S06_RS06120	Nucleoid-associated protein	−1.06	0.00	0.03
		C7S06_RS06180	Nitronate monooxygenase family protein	−1.48	0.00	0.02
		C7S06_RS13545	Glutamate dehydrogenase	−2.85	0.00	0.01
		C7S06_RS06985	Hemolysin III family protein	−2.22	0.00	0.00
		C7S06_RS07095	23S ribosomal RNA	−8.91	0.00	0.00
		C7S06_RS07110	16S ribosomal RNA	−9.86	0.00	0.00
		C7S06_RS07485	PepSY domain-containing protein	1.82	0.00	0.02
		C7S06_RS07550	GrxB family glutaredoxin	−1.80	0.00	0.01
		grpE	Nucleotide exchange factor GrpE	−1.54	0.00	0.01
		C7S06_RS08065	NAD(*P*)H-dependent oxidoreductase	−3.44	0.00	0.01
		xth	Exodeoxyribonuclease III	−1.15	0.00	0.03
		C7S06_RS08615	Metalloregulator ArsR/SmtB family transcription factor	−1.89	0.00	0.00
		C7S06_RS08785	Hypothetical protein	−2.32	0.00	0.00
		C7S06_RS08795	immunity 41 family protein	−4.78	0.00	0.00
		C7S06_RS09345	23S ribosomal RNA	−8.95	0.00	0.00
		C7S06_RS09360	16S ribosomal RNA	−9.92	0.00	0.00
		C7S06_RS09460	chloride channel protein	1.18	0.00	0.04
		C7S06_RS09850	23S ribosomal RNA	−8.91	0.00	0.00
		C7S06_RS09865	16S ribosomal RNA	−9.94	0.00	0.00
		C7S06_RS10210	Pilin	−6.01	0.00	0.02
		C7S06_RS10410	tRNA-Met	−6.95	0.00	0.01
		ssrS	6S RNA	−5.32	0.00	0.00
		C7S06_RS10580	Amino acid ABC transporter permease	2.03	0.00	0.01
		C7S06_RS10585	Amino acid ABC transporter permease	1.76	0.00	0.02
		C7S06_RS10775	Hypothetical protein	−1.77	0.00	0.01
		C7S06_RS11300	16S ribosomal RNA	−9.87	0.00	0.00
		C7S06_RS11315	23S ribosomal RNA	−8.94	0.00	0.00
		C7S06_RS11330	Helix-hairpin-helix domain-containing protein	−1.28	0.00	0.02
		C7S06_RS12160	tRNA-Asp	−9.42	0.00	0.00
		C7S06_RS12240	MFS transporter	1.83	0.00	0.00
Condition 4	All Control (n = 3) vs All Tolerant (n = 12) isolates	C7S06_RS01850	tRNA-Ser	−6.10	0.00	0.00
		C7S06_RS02470	IS1595 family transposase	−2.46	0.00	0.02
		C7S06_RS02975	tRNA-Gln	−3.89	0.00	0.02
		C7S06_RS03100	tRNA-Ser	−7.28	0.00	0.00
		C7S06_RS04945	tRNA-Leu	−4.95	0.00	0.02
		C7S06_RS04950	tRNA-Ser	−5.38	0.00	0.00
		C7S06_RS06055	Hypothetical protein	−2.54	0.00	0.02
		C7S06_RS10410	tRNA-Met	−5.07	0.00	0.02
		ssrS	6S RNA	−3.38	0.00	0.02
		C7S06_RS12165	tRNA-Val	−3.75	0.00	0.02

^*a*^ NA- not available.

**Fig 3:**
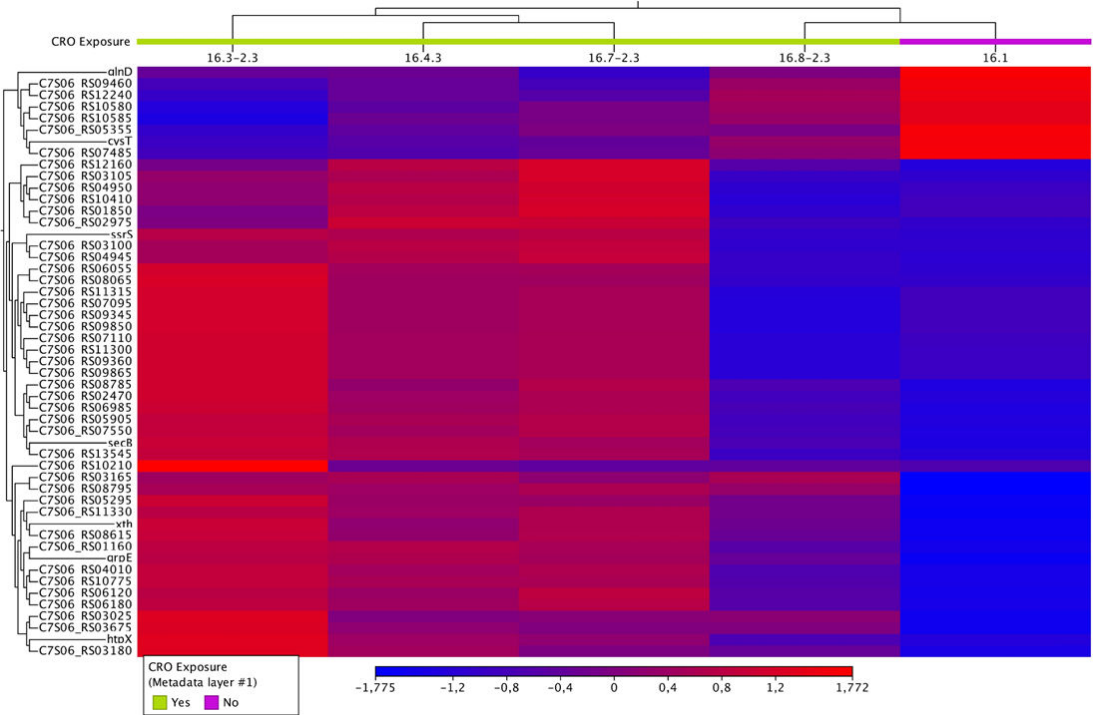
Heatmap of differentially expressed genes in condition 3 showing the expression levels of all 51 DEGs across the CRO-tolerant and control isolates

In condition 3, a total of 51 DEGs were identified, and most were predominantly downregulated. Significant downregulation was observed in genes such as *secB* (Protein-export chaperone SecB) and C7S06_RS01850 (tRNA-Ser), with log_2_ fold changes of −1.85 and −7.99, respectively. The downregulated genes also included four copies of 23S rRNA, 6S RNA, tRNAs (*n* = 7), hypothetical protein (*n* = 5), and pilin (Fig. 3 and Table 2). Out of the 51 DEGs, eight genes were upregulated with log_2_ fold changes ranging between 1.13 and 2.03. These included specific transporter genes, such as *cysT* (sulfate ABC transporter permease subunit), *glnD* ([protein-PII] uridylyltransferase), C7S06_RS05355 (sulfate ABC transporter substrate-binding protein), C7S06_RS07485 (PepSY domain-containing protein), C7S06_RS09460 (chloride channel protein), two amino acid ABC transporter permeases (C7S06_RS10580 and C7S06_RS10585), and C7S06_RS12240 (MFS transporter).

**Fig 4:**
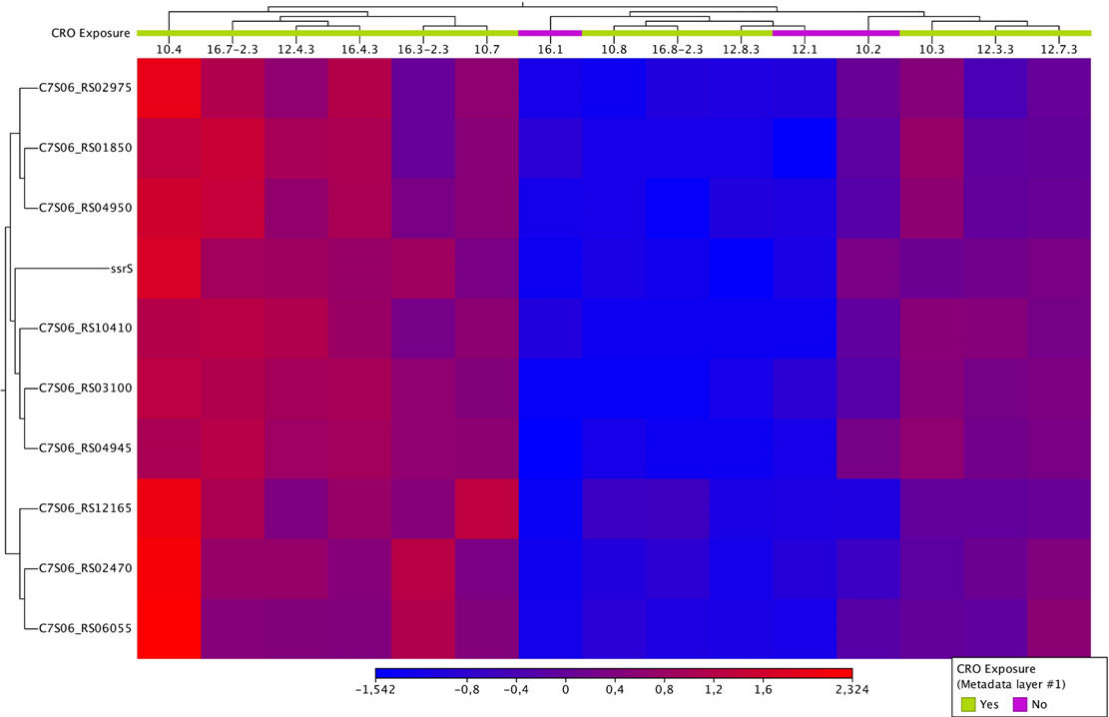
Heatmap of differentially expressed genes in condition 4 showing the expression levels of all 10 DEGs across the CRO-tolerant and control isolate

In condition 4, 10 DEGs were identified, including tRNAs, ssrS, and a hypothetical protein that were all significantly downregulated (Table 2, Fig. 4).

## DISCUSSION

Previously, we reported that tolerance to CRO can be induced in a CRO-susceptible (<0.004 µg/mL) *N. gonorrhoeae* WHO P reference isolate (24). In the current analysis, we describe the differentially expressed genes using RNA-Seq for CRO-tolerant isolates (*n* = 12). The DEGs identified in the various conditions underscore the complexity of bacterial adaptation during ceftriaxone tolerance.

No DEGs were identified in condition 1, when isolates had only been exposed to CRO for one day, suggesting that early response to antibiotic exposure may not involve significant transcriptional changes. In condition 2, following 3 days of CRO exposure, 13 DEGs were identified, all of which were significantly downregulated. Downregulation of tRNA genes (e.g., tRNA-Ser, tRNA-Leu), 16S and 23S ribosomal RNA genes suggests a decrease in overall bacterial protein synthesis. The abundance of individual tRNA varies with growth rate and reflects the codon frequencies in the corresponding mRNA pools (31). However, when translation demand decreases due to stress reponse—e.g., reduced mRNA availability—tRNAs become susceptible to degradation by ribonucleases, thus preventing the accumulation of uncharged tRNAs (32). Thus, the downregulation of tRNAs in CRO-tolerant *N. gonorrhoeae* could be a survival strategy where the bacteria can optimize their metabolic activity to survive under high antibiotic pressure.

Bacterial ssrS (6S RNA), a small non-coding RNA that is conserved in all bacteria, inhibits transcription via direct binding to RNA polymerase holoenzymes (33, 34). 6S RNA has been shown to be a key player in stress responses; for example, in *E. coli,* it is involved in oxidative stress response (35, 36). Deletion of 6S RNA in *Bacillus thuringiensis* results in impaired growth in the stationary phase (37). Downregulation of ssrS in conditions 2 to 4 may reflect a regulatory shift aimed at potentially allowing selective reactivation of transcriptional factors in response to intermittent antimicrobial exposure. However, the precise functional consequences of ssrS downregulation in *N. gonorrhoeae* remain to be elucidated.

In condition 3, after 7 days of CRO exposure, the transcriptomic landscape revealed 51 DEGs, indicating a more extensive bacterial response. The downregulation of additional genes, including *secB* (Protein-export chaperone SecB), suggests less need for these systems during stress as chaperones play important roles in withstanding stress associated with intracellular survival (38, 39). Interestingly, in condition 3, the upregulation of specific transporter genes, such as *cysT* (Sulfate ABC transporter permease subunit) and *glnD* ([protein-PII] uridylyltransferase), was observed. Microorganisms require sulfur for growth, and in *E. coli* and *Salmonella typhimurium,* the ABC-type sulfate transporter, which is responsible for the transport of inorganic sulfate into microbial cells, consists of the periplasmic sulfate binding protein (Sbp), which interacts with the permease components CyT and CysW (40). The upregulation of *cysT* in *N. gonorrhoeae* suggests that it helps maintain sulfur homeostasis, which is vital for bacterial survival and adaptation. The primary sensor in many bacterial nitrogen signal transduction (NSR) systems is the GlnD protein, a large multidomain uridylyltransferase and uridylyl cleavage enzyme whose primary uridylylation targets are the PII proteins, GlnB, and GlnK (41). The uridylylation state of these proteins is regulated by α-ketoglutarate and glutamine so that when GlnD senses that there is not enough nitrogen available to synthesize adequate glutamine, it activates the NSR by uridylylating the PII proteins (42–44). Thus, the upregulation of *glnD* may reflect GlnD’s uridylylation of GlnB and GlnK, which in turn regulates the expression of genes involved in nitrogen uptake and assimilation. However, in our data set *glnA* and *glnE* were not significantly differentially expressed. This suggests that *glnD* upregulation may play a broader, yet undefined and possibly non-canonical role in stress adaptation or ceftriaxone tolerance, beyond its canonical function in nitrogen signalling. Notably, several genes that were differentially expressed in condition two were not significantly altered in condition 3, despite both groups having undergone the same ceftriaxone exposure protocol. As these conditions represent independent lineages, the differences likely reflect lineage-specific transcriptional responses to CRO-induced stress. Thus, the emergence of ceftriaxone tolerance may involve multiple, potentially divergent regulatory pathways.

In condition 4, where all the control samples (*n* = 3) were compared to all the tolerant samples (*n* = 12) across multiple time points, 10 DEGs were consistently identified, including downregulated tRNA genes and ssrS (6S RNA). This consistent downregulation across different conditions and time points suggests that these genes are central to the bacterial response to CRO exposure and may represent core components of the tolerance mechanism. Of note, two hypothetical proteins with no assigned function were identified, illustrating how much remains to be learned about tolerance in *Neisseria* spp.

The caveats of the study include the small sample sizes and the fact that the transcriptomic data were derived from *in vitro* experiments; as such, the findings may not entirely reflect the gene expression patterns that occur *in vivo* (20). The findings were not validated using RT-qPCR validation, and some transcriptomic changes observed after 7 days of exposure may reflect broader stress adaptation rather than tolerance-specific mechanisms. Future studies should include targeted validation of key transcripts using RT-qPCR. Gene knockout experiments need to be conducted to validate the roles of the identified DEGs in ceftriaxone tolerance. The observed downregulation of ribosomal genes could be due to the expression bias introduced during the ribodepletion of rRNA. Finally, although we did not quantify the growth rates of tolerant versus non-tolerant isolates, the tolerant isolates consistently exhibited slower colony formation, suggestive of reduced growth rate (Kanesaka et al., unpublished). Future studies should include quantitative assessments using *in vitro* growth curves.”

Further research could evaluate if similar transcriptomic changes occur during human infections. It will also be important to assess if the probability and timing of the emergence of tolerance vary by anatomical site of infection and exposure to various antimicrobials. We have not assessed the prevalence of tolerance in pharyngeal infections. This will be useful to do as infections at this site are more difficult to eradicate and have been frequently implicated in the emergence of antimicrobial resistance (45, 46).

Future directions should involve using *in vivo* animal models or clinical isolates from patients to validate the relevance of the identified DEGs. This is particularly important given the studies that have found that tolerance facilitates the emergence of AMR (11), as well as our recent finding that anorectal *N. gonorrhoeae* infections showed a higher prevalence of tolerance compared to urethral infections (26). Thus, tolerance may partially explain why a higher proportion of anorectal infections are asymptomatic (26).

In conclusion, this exploratory study provides preliminary insights into the molecular basis of antibiotic tolerance. In general, tolerance was associated with the downregulation of central protein synthesis and later upregulation of genes encoding transporter proteins.
